# Material Removal Mechanism and Performance Evaluation of Focused Ultrasonic-Assisted Abrasive Waterjet Polishing (FUAP) of Monocrystalline Silicon

**DOI:** 10.3390/ma19153339

**Published:** 2026-08-05

**Authors:** Kun Ren, Julong Yuan, Hua Li, Qing Miao, Zhongwang Wang, Qing Liu, Xiang Liu

**Affiliations:** 1 School of Mechanical Engineering, Suzhou University of Science and Technology, Suzhou 215009, China; renkun@usts.edu.cn (K.R.); miaoqing@usts.edu.cn (Q.M.);; 2 Suzhou Key Laboratory of Precision and Efficient Processing Technology, Suzhou 215009, China; 3 College of Mechanical Engineering, Zhejiang University of Technology, Hangzhou 310023, China

**Keywords:** monocrystalline silicon, focused ultrasonic vibration, waterjet polishing, removal mechanism, process analysis

## Abstract

Hard and brittle material components with complex curved surfaces are widely used in critical foundational parts within aerospace, optoelectronics, and other fields. Their machining quality directly determines the performance and reliability of high-end equipment. However, the inherent properties of hard and brittle materials make them prone to surface/subsurface damage during traditional polishing processes, and maintaining the form accuracy of complex curved surfaces is challenging. Although abrasive waterjet polishing enables non-contact flexible processing, its energy efficiency is low. Additionally, although ultrasonic-assisted polishing can improve material removal, its spatial localization is insufficient, limiting energy utilization efficiency. To address these issues, this paper proposes a novel method of focused, ultrasonic, vibration-assisted abrasive waterjet polishing. The influence of the radiation force and cavitation force of the focused ultrasonic field on abrasive particle motion is analyzed, and analytical equations for abrasive particle velocity are established. Subsequently, single-factor and response surface methodologies are employed to systematically evaluate the influence of process parameters on machining quality and efficiency. The material removal process during FUAP involves both plastic shearing/chip formation and localized brittle fracture. Focused ultrasonic assistance promotes micro-cutting and plastic shearing, while localized crushing pits indicate that brittle fracture remains non-negligible. The focused ultrasound superimposes alternating stress onto the impact action, mitigating microscale crushing pit defects during the brittle removal process of monocrystalline silicon. Furthermore, appropriately increasing ultrasonic power, enlarging abrasive particle size, and raising abrasive concentration all contribute to enhanced material removal from monocrystalline silicon. Adjusting the nozzle height to the effective region of the focused ultrasonic energy field promotes material removal via chip formation while avoiding pit defects caused by excessive fracture. These results suggest that focused ultrasonic energy can be effectively integrated into abrasive waterjet polishing to enhance material removal while suppressing brittle surface defects, thereby offering a promising strategy for the ultra-precision finishing of hard and brittle components with complex curved surfaces.

## 1. Introduction

Hard and brittle materials (such as monocrystalline silicon, optical glass, engineering ceramics, etc.) are widely used in the manufacturing of critical components like integrated circuit substrates, optical lenses, and space mirrors due to their excellent properties such as high hardness, wear resistance, high-temperature resistance, and corrosion resistance [1,2,3]. Concurrently, the demands for surface quality and integrity of complex curved surfaces and thin-walled components, including aero-engine blades, complex mold cavities, hemispherical resonator assemblies, and aspheric lenses, are becoming increasingly stringent. Polishing remains virtually the only method to achieve ultra-high surface quality [4,5,6]. Therefore, achieving efficient, high-quality polishing of hard and brittle materials and complex curved surface parts has become a critical issue urgently needing resolution in the field of precision manufacturing.

To address these demands, researchers have developed various methods based on traditional mechanical polishing [7,8,9], including Chemical Mechanical Polishing (CMP) [10], Ultrasonic Vibration-Assisted Polishing (UVAP) [11], Magnetorheological Finishing (MRF) [12], Shear-Thickening Polishing (STP) [13], and Abrasive Flow Polishing (AFP) [14], achieving good results under specific conditions. However, these methods have limitations regarding efficiency, surface quality, equipment cost, and adaptability to complex curved surfaces: CMP is primarily suitable for flat surfaces; MRF requires a magnetic medium and an external magnetic field, making the equipment complex and costly [15]; AFP efficiency is limited by the uniformity and stability of the abrasive flow field. These shortcomings restrict the high-quality polishing of complex curved surface parts made of hard and brittle materials, necessitating the exploration of new polishing principles and methods that balance high precision, high efficiency, and low cost.

Ultrasonic vibration-assisted abrasive flow polishing utilizes ultrasonic vibration to propel abrasive particles in the fluid to impact the workpiece surface, enabling microscale material removal. Ralchenko et al. [16] used 22 kHz longitudinal ultrasonic vibration with a diamond micro-powder slurry, reducing the surface roughness of a diamond film from Ra 5 μm to Ra 0.5 μm within 5 min. Liang et al. [17] combined an ultrasonic Fenton method with chemical magnetorheological polishing, significantly improving the efficiency of oxidation layer generation and polishing quality of a GaN surface within 60 min. He et al. [18] investigated electrophoresis-assisted micro-ultrasonic machining (EPAMUSM) and found its edge chipping (0.03) to be much smaller than that of conventional micro-ultrasonic machining (0.22), with a slightly better material removal rate. Huang Weiqing et al. [19] revealed the critical condition for the brittle–ductile transition of sapphire through abrasive impact simulations and experiments; when the abrasive particle size was 1.5 μm, the surface roughness decreased from 1.049 μm to 0.052 μm. Qi et al. [20] utilized ultrasonic levitation to form a standing wave acoustic field, reducing the surface roughness of a silicon wafer from Ra 45.99 nm to Ra 8.99 nm. Lv Zhe, Hou Rongguo, and colleagues [21,22] systematically studied the effects of different ultrasonic vibration modes (nozzle ultrasound, workpiece normal/tangential/torsional ultrasound) on the polishing of alumina ceramics and glass, finding that ultrasonic vibration enhances the plastic shearing removal capability of abrasives and improves erosion textures. Ge et al. [23] proposed a photochemically assisted pulsed jet polishing method, combining the Johnson–Cook constitutive model with finite-element-smoothed particle hydrodynamics simulation, achieving an approximately 20% increase in material removal rate and about 33.9% improvement in surface roughness. Li et al. [24] employed ultrasonic-coupled abrasive jet polishing, reducing the surface roughness from 3.2 μm to 69 nm at an amplitude of 20 μm. These studies demonstrate the significant potential of ultrasonic vibration-assisted polishing for hard and brittle materials, but existing methods still face challenges such as low ultrasonic energy utilization, insufficient adaptability to curved surfaces, and difficulty in balancing processing efficiency with surface quality.

To overcome these bottlenecks, some researchers have explored the application of focused ultrasound technology. High-Intensity Focused Ultrasound (HIFU) is well-established in medical therapy and ultrasonic testing, using acoustic lenses to concentrate ultrasonic energy at a focal point, generating high-intensity mechanical, cavitation, and thermal effects [25]. Beaucamp et al. [26] were the first to introduce focused ultrasound into the polishing field, using a 26–130 kHz longitudinal vibration transducer and an acoustic lens to focus the polishing slurry, achieving cavitation jet-assisted polishing on BK7 glass and electroless nickel. At the same surface quality, the processing efficiency was 380% that of polishing without ultrasound. This result indicates that focused ultrasound can significantly enhance ultrasonic energy utilization and enable efficient polishing of curved workpieces at relatively long distances, offering a new approach for polishing hard and brittle materials with complex surfaces. Although the above studies have demonstrated the potential of ultrasonic-assisted polishing and focused ultrasonic polishing, several issues remain insufficiently addressed. First, most ultrasonic-assisted abrasive polishing methods rely on non-focused ultrasonic vibration applied to the nozzle, workpiece, or fluid medium. The ultrasonic energy is therefore dispersed over a relatively large region, which limits local energy utilization efficiency and reduces the controllability of abrasive particle motion. Second, existing focused ultrasonic polishing studies have mainly verified the feasibility of using focused acoustic energy to improve polishing efficiency, whereas the coupled effects of acoustic radiation force, cavitation micro-jets, and cavitation shock waves on abrasive particle motion have not been sufficiently modeled. Third, the material removal behavior of monocrystalline silicon under FUAP remains unclear, especially the competition between plastic chip formation and brittle fracture. Therefore, a more detailed understanding of the removal mechanism and process parameter effects is required for applying focused ultrasonic energy to the ultra-precision polishing of hard and brittle materials.

Therefore, a FUAP method is proposed in this study to improve the energy utilization and polishing performance of abrasive waterjet polishing for hard and brittle materials. The present work aims to clarify the influence of focused ultrasonic energy on abrasive particle motion through acoustic radiation force and cavitation effects. The dominant material-removal mechanism of monocrystalline silicon under the coupled action of abrasive impact, focused ultrasonic vibration, and cavitation is then investigated. The effects of key process parameters, including ultrasonic power, abrasive size, abrasive concentration, and jet angle, on surface roughness, surface morphology, and material removal rate are systematically evaluated. Particular attention is paid to the role of the effective focused ultrasonic energy region in promoting chip-formation-dominated material removal while suppressing brittle-fracture defects. To achieve these objectives, an abrasive-particle motion model and a material removal model are established, followed by single-factor polishing experiments on monocrystalline silicon. The results are expected to provide theoretical and technical support for the ultra-precision polishing of hard and brittle components with complex curved surfaces.

## 2. Establishment of Material Removal Model for Monocrystalline Silicon Under Focused Ultrasonic Vibration Conditions

### 2.1. Radiation Force of the Focused Ultrasonic Field

A focused ultrasonic transducer, fabricated using a partially concave spherical surface, can generate spherical wavefronts. Using a custom-designed concave spherical self-focusing ultrasonic vibration transducer, applying geometrical acoustics methods, and combining ultrasonic vibration theory with Beissner and Rayleigh analysis [27,28], the formula for calculating the radiation force in the focused ultrasonic field can be derived. It should be noted that the following assumptions were made during the calculation of the focused ultrasonic radiation force [29]:

(1) The concave spherical device surface produces an ideal focused sound field, i.e., uniform sound intensity.
(2) Energy loss during ultrasonic transmission in water is neglected.
(3) When the ultrasonic wave passes through the focal region, the ultrasonic energy flux is constant.

The sound pressure *p* within the radiation field of any source is calculated by [30]:

(1) $$p=if\rho \iint_{S}u(R)\frac{e^{-jkr}}{r}ds$$

where *ρ* and *f* are the density of the transmission medium (water) and the ultrasonic vibration frequency, respectively; *k* is the angular wave number (*k =* 2*πf*/*c*), with *c* being the speed of sound in water; *R* is the position vector from the coordinate origin to any point on the ultrasonic radiation surface; *r* is the position vector from any point on the radiation surface to a point in the sound field; *S* represents the radiation surface of the concave spherical focused ultrasonic transducer; and *ds* is the area element of the radiation surface.

**Figure 1:**
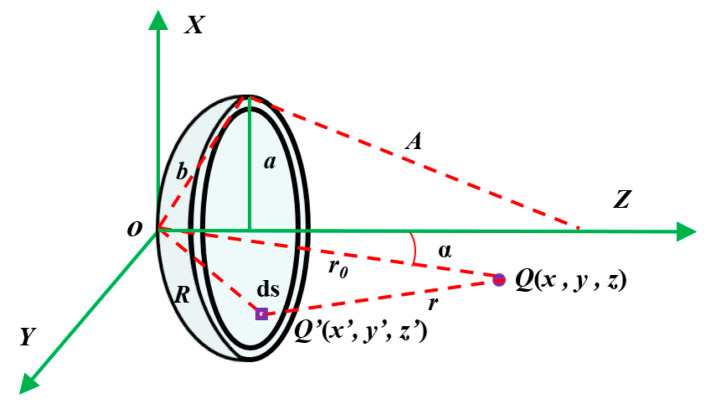
The geometric shape and calculated positional relationship of concave spherical surface focused ultrasonic vibration transducer.

To facilitate theoretical analysis of the acoustic field characteristics of the focused ultrasonic transducer, the concave spherical surface is simplified, and a coordinate system is established as shown in Figure 1. Let *a* be the aperture radius of the concave spherical transducer, *b* be the distance from the edge of the aperture to the center, *r*_0_ be the distance from the coordinate origin (i.e., the center vertex of the concave spherical transducer) to any point *Q*(*x*, *y*, *z*) in the ultrasonic field, and *α* be the angle between *r*_0_ and the Z-axis. *Q*’(*x’*, *y’*, *z’*) represents the coordinates of any point on the area element ds, and *β* is the angle between the line perpendicular to the *Z*-axis through point *Q* projected onto the *XOZ* plane and the line perpendicular to the *Z*-axis through point *Q’* projected onto the *XOZ* plane.

The theoretical formulas for the sound pressure distribution *p*(*r*) on the plane *Z* = *A* and the sound pressure distribution *p*(*Z*) along the acoustic axis can be derived:

(2) $$p(r)=f\rho u_{0}\left(\frac{2\pi^{2}a^{2}}{A\lambda }\right)\times \left[\frac{2J_{1}(kar/A)}{kar/A}\right]$$

(3) $$p(Z)=f\rho u_{0}\left(\frac{2\pi^{2}a^{2}}{A\lambda }\right)\times \frac{\sin\left[\frac{\pi a^{2}}{2Z\lambda }\left(\frac{Z}{A}-1\right)\right]}{\frac{\pi a^{2}}{2Z\lambda }\left(\frac{Z}{A}-1\right)}$$

where *λ* is the acoustic wavelength, and *J*_1_ is the first-order Bessel function.

### 2.2. Focused Ultrasonic Cavitation Effect

**Figure 2:**
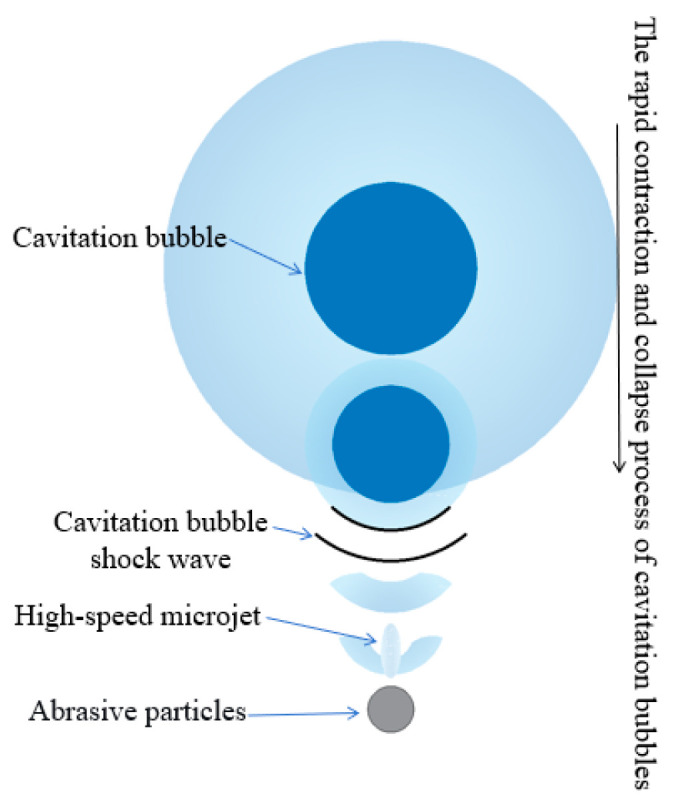
Schematic of cavitation-induced microjet and shock wave acting on abrasive particles in the focused ultrasonic field.

During liquid flow, the disturbance caused by ultrasonic vibration leads to drastic local pressure changes within the liquid. Due to the periodic nature of ultrasonic vibration, when the pressure around a cavitation bubble increases sharply, the bubble collapses rapidly, generating localized instantaneous high temperatures and high pressures, resulting in high-speed micro-jets and intense shock waves externally. Consequently, the abrasive flow in the focused ultrasonic energy field is also strongly affected by ultrasonic cavitation (Figure 2).

(1) High-speed Microjet

Under high-frequency ultrasonic vibration, when a cavitation bubble moves with the flow to the rear of an abrasive particle, the water flow at the bubble’s front edge is obstructed by the particle. The rear edge of the bubble, being in a free state, reacts more sensitively than the front edge, leading to rapid contraction and collapse. Therefore, the cavitation bubble behind the particle collapses instantaneously, impacting the water flow and forming a microjet that acts on the rear of the particle, propelling it forward. The formula for the microjet velocity is as follows [31,32]:

(4) $$v_{jet}=8.97{\left(\frac{H}{R_{CB}}\right)}^{2}\sqrt{\frac{P_{\infty }-P_{V}}{\rho }}$$

(5) $$P_{jet}=v_{jet}\frac{\rho_{s}c_{s}\rho_{a}c_{a}}{\rho_{s}c_{s}+\rho_{a}c_{a}}$$

where *R_CB_* is the diameter of the cavitation bubble, *P_V_* is the saturated vapor pressure inside the bubble, *P_∞_* is the ambient pressure, *V_jet_* is the high-speed microjet velocity, *P_jet_* is the pressure generated by the water hammer effect of the microjet, and *ρ* and *c* represent density and ultrasonic propagation speed (subscript *s* denotes the transmission medium water, and subscript *a* denotes the abrasive particle).

(2) Intense Shock Wave

Unlike the high-speed microjet, the rapid contraction and expansion of cavitation bubbles under continuous ultrasonic vibration generate shock waves behind the particle. Vogel [33] analyzed the relationship between shock wave velocity and particle motion velocity, deriving the theoretical formula for the shock wave pressure *P_s_*:

(6) $$P_{s}=k_{1}\rho u_{s}\left(10^{(u_{s}-c_{s})/k_{2}}-1\right)+P_{\infty }$$

(7) $$u_{a}=k_{1}\left(10^{(u_{s}-c_{s})/k_{2}}-1\right)$$

(8) $$u_{s}=c_{s}+k_{2}\mathrm{lg}\left(\frac{u_{a}}{k_{1}}+1\right)$$

(9) $$P_{\infty }=P_{s}\sin(2\pi ft)+P_{0}$$

where *u_s_* is the shock wave velocity, *u_a_* is the velocity of the abrasive particle before being impacted by the shock wave, *c_s_* is the speed of sound in water, *P*_0_ is the static ambient pressure, and *k*_1_ and *k*_2_ are velocity fitting correlation coefficients, typically taken as *k*_1_ = 5190 m/s and *k*_2_ = 23,056 m/s.

### 2.3. Force Model on Abrasive Particles in the Focused Ultrasonic Field

**Figure 3:**
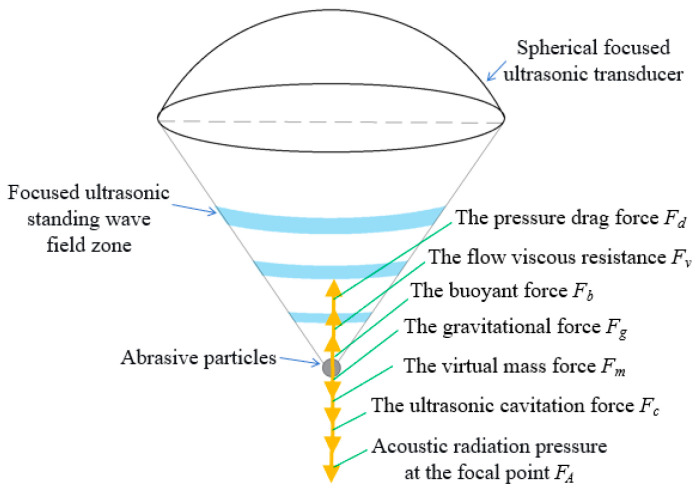
Force model of abrasive particles in the focused ultrasonic energy field.

As shown in Figure 3, the forces acting on a single abrasive particle in the waterjet within the ultrasonic field include the pressure at the focal point of the focused acoustic field *F_A_*, the pressure drag force *F_d_*, the flow viscous resistance *F_v_*, the buoyant force *F_b_*, the gravitational force *F_g_*, the ultrasonic cavitation force *F_c_*, and the virtual mass force *F_m_*. The ultrasonic cavitation force *F_c_* consists of the cavitation microjet force *F_jet_* and the cavitation shock wave force *F_s_*. Based on the force balance on the abrasive particle in the focused ultrasonic field [34], the particle’s equation of motion is formulated as follows:

(10) $$\sum F=F_{g}+F_{c}+F_{m}+F_{A}-\left(F_{d}+F_{v}+F_{b}\right)=m_{a}\frac{dv_{a}}{dt}$$

(11) $$F_{c}=F_{jet}+F_{s}$$

(12) $$F_{A}=S_{a}\times p(A)$$

(13) $$p(A)=f\rho \left[P_{a}\sin(2\pi ft+\psi )\right]\cdot \left(\frac{2\pi^{2}a^{2}}{A\lambda }\right)\cdot \frac{\sin\left[\frac{\pi a^{2}}{2Z\lambda }\left(\frac{Z}{A}-1\right)\right]}{\frac{\pi a^{2}}{2Z\lambda }\left(\frac{Z}{A}-1\right)}$$

where *S_a_* is the force-bearing area of the abrasive particle, *p*(*A*) is the sound pressure value when the geometric focal length of the transducer equals the acoustic focal length of the radiating surface, *P_a_* is the amplitude, *t* is time, *ψ* is the initial phase, and *v_a_* is the particle velocity without the superimposed waterjet velocity.

### 2.4. Abrasive Particle Velocity Under the Focused Ultrasonic Field

The solution equation for the abrasive particle velocity under the focused ultrasonic field can be derived as:

(14) $$\begin{array}{l} m_{a}g+v_{jet}\frac{\rho_{s}c_{s}\rho_{a}c_{a}}{\rho_{s}c_{s}+\rho_{a}c_{a}}+k_{1}\rho u_{s}\left(10^{(u_{s}-c_{s})/k_{2}}-1\right)\\ +P_{\infty }-0.5v_{a}\rho_{s}\frac{dv_{a}}{dt}+S_{a}\cdot f\cdot \rho \cdot \left[P_{a}\sin(2\pi ft+\psi )\right]\cdot \left(\frac{2\pi^{2}a^{2}}{A\lambda }\right)\cdot \frac{\sin\left[\frac{\pi a^{2}}{2Z\lambda }\left(\frac{Z}{A}-1\right)\right]}{\frac{\pi a^{2}}{2Z\lambda }\left(\frac{Z}{A}-1\right)}\\ -0.5k_{3}\rho_{s}S_{a}v_{a}^{2}-6\pi \mu r_{a}v_{a}-\rho_{s}gV_{a}=m_{a}\frac{dv_{a}}{dt} \end{array}$$

From the above equation, it can be seen that it comprehensively considers multiple factors such as focused ultrasonic field radiation force, abrasive parameters, ultrasonic cavitation force, and ultrasonic vibration parameters.

### 2.5. Material Removal Model for Focused Ultrasonic Vibration-Assisted Abrasive Waterjet Polishing of Monocrystalline Silicon

Abrasive particles in the focused ultrasonic-assisted waterjet field move at relatively high speeds, and the material removal process from the workpiece generally includes both brittle and plastic removal. This depends on the energy of the particle impacting the workpiece. During brittle removal, the instantaneous impact pressure first causes brittle cracks on the workpiece surface, which propagate and lead to material fragmentation and detachment from the substrate, leaving damage defects like pits on the subsurface. During plastic removal, under the high-speed impact and shearing action of the abrasive particles, the workpiece material undergoes yield failure and is stripped from the substrate.

Based on Hertz contact theory, the mechanism of abrasive particle penetration into the hard and brittle workpiece to form chips is analyzed. Assuming the abrasive particle is spherical, with radius *r_a_*, volume *V_a_*, and mass *m_a_*, the load it bears when penetrating to a depth *h* is *F*, and the impact angle is *θ*. The relationship between penetration depth *h* and particle radius *r_a_* is:

(15) $$h^{3}=\frac{9}{16}\cdot \frac{F^{2}}{r_{a}}{\left(\frac{1-\mu_{a}^{2}}{E_{a}}+\frac{1-\mu_{w}^{2}}{E_{w}}\right)}^{2}$$

where *μ_a_* and *E_a_* are Poisson’s ratio and elastic modulus of the abrasive, and *μ_w_* and *E_w_* are Poisson’s ratio and elastic modulus of the workpiece. Consequently:

(16) $$h=1.73r_{a}^{0.5}\cdot v^{0.8}\cdot \rho_{a}^{0.4}{\left(\frac{1-\mu_{a}^{2}}{E_{a}}+\frac{1-\mu_{w}^{2}}{E_{w}}\right)}^{0.4}$$

The ductile-regime removal of monocrystalline silicon is closely related to the penetration depth of abrasive particles. When the penetration depth is lower than the critical penetration depth, plastic deformation and chip formation are more likely to occur. When the penetration depth exceeds the critical value, median and lateral cracks can be induced, leading to brittle fracture. Therefore, the critical penetration depth *h_c_* was introduced to evaluate the removal mode of monocrystalline silicon:

(17) $$h_{c}=\eta \frac{E_{w}}{H_{w}}{\left(\frac{K_{IC}}{H_{w}}\right)}^{2}$$

where *E_w_*, *H_w_*, and *K_IC_* are the elastic modulus, hardness, and fracture toughness of monocrystalline silicon, respectively. *η* is an empirical coefficient, which is usually taken as 0.15 for brittle–ductile transition estimation.

According to the material properties listed in Table 1, the critical penetration depth of monocrystalline silicon was approximately 9.2 nm under the adopted material parameters.

Combining with the conical volume formula, the volume of material removed from the brittle monocrystalline silicon workpiece by a single abrasive particle during focused ultrasonic vibration-assisted abrasive waterjet polishing is:

(18) $$V_{brittle}=\frac{1}{3}\pi r_{c}^{2}h=3.13r_{a}^{2}\cdot v^{1.6}\rho_{a}^{0.8}{\left(\frac{1-\mu_{a}^{2}}{E_{a}}+\frac{1-\mu_{w}^{2}}{E_{w}}\right)}^{0.8}$$

### 2.6. Acoustic Pressure Simulation of the FUAP Nozzle Assembly

**Figure 4:**
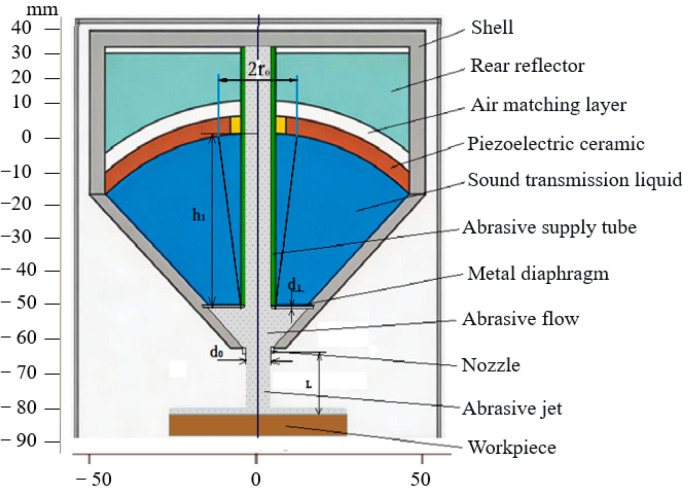
Two-dimensional axisymmetric frequency-domain acoustic field model.

To avoid using the hydro-acoustic equations only as qualitative descriptions, a two-dimensional axisymmetric acoustic-pressure simulation was performed using COMSOL Multiphysics 6.0. The simulation was used to evaluate the focused acoustic pressure field in the FUAP nozzle assembly and to verify whether the ultrasonic energy could be concentrated near the nozzle outlet. The simulation was conducted by solving the homogeneous Helmholtz equation in cylindrical coordinates using the pressure acoustics module in the frequency domain. The simulation model is shown in Figure 4.

The FUAP polishing head consists of a concave spherical piezoelectric ceramic ultrasonic transducer, a rear reflector, a rear matching layer, a front matching layer, a metallic diaphragm, a front conical chamber, and a conical nozzle. The metallic diaphragm separates the clean-water chamber from the abrasive-slurry chamber. During operation, the upper chamber is filled with clean circulating water, while the abrasive slurry is supplied into the lower conical chamber through the central feeding tube. Therefore, focused ultrasonic vibration is transmitted through the water–metallic diaphragm–abrasive slurry path before being concentrated near the nozzle region. In the model, *h*_1_ represents the distance from the vertex of the concave spherical piezoelectric ceramic transducer to the metallic diaphragm, *d*_1_ represents the diaphragm thickness, and *d*_0_ represents the nozzle diameter. The quarter-wavelength and half-wavelength positions in the acoustic transmission medium are denoted *λ*_1/4_. The main simulation parameters are listed in Table 2. The PZT-8 concave spherical shell had an aperture radius of 45 mm, a thickness of 5 mm, and a spherical radius of 65.25 mm. The rear reflector was made of 45 steel with a spherical radius of 69 mm. The rear matching layer and front matching layer were air and silicone rubber, respectively. The metallic diaphragm was made of 316L stainless steel with a thickness of 0.1 mm. The vibration amplitude and frequency were 0.5 μm and 430 kHz, respectively.

**Figure 5:**
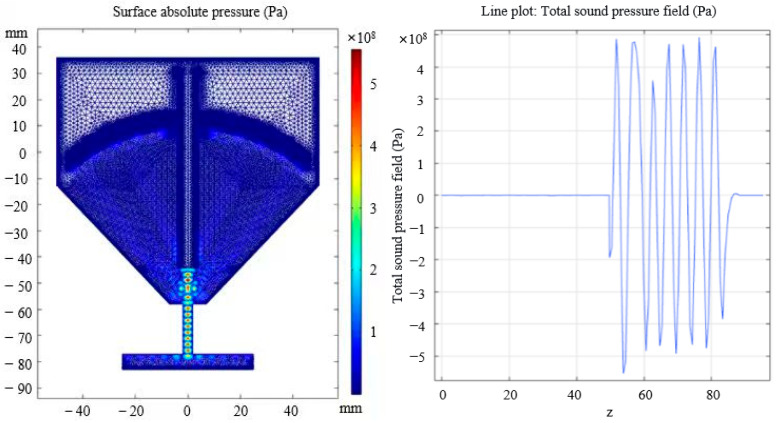
One-quarter-wavelength sound pressure distribution and sound pressure curve (*h*_1_ = 57λ_1/4_).

**Figure 6:**
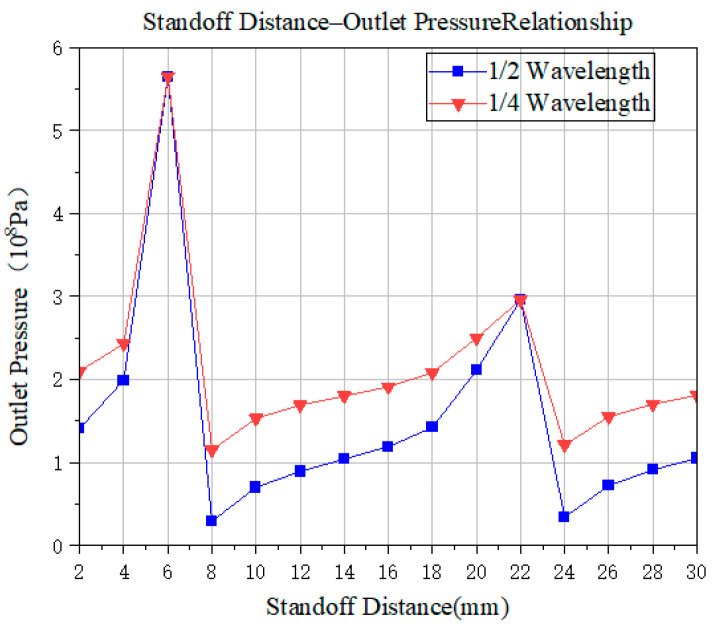
Outlet pressure at different standoff distances.

The metallic diaphragm plays two roles in the FUAP nozzle assembly. First, it separates the clean circulating water from the abrasive slurry. Second, it transmits ultrasonic vibration from the water chamber to the abrasive-slurry chamber. A thinner diaphragm is beneficial for reducing acoustic energy dissipation. However, an excessively thin diaphragm may suffer from insufficient stiffness and deformation. Therefore, a 0.1 mm thick 316L stainless-steel diaphragm was selected in the present simulation. The influence of diaphragm position, standoff distance, and nozzle diameter on the simulated outlet acoustic pressure was then analyzed. As shown in Figure 5 and Figure 6, the acoustic pressure was concentrated near the nozzle region when the diaphragm was placed at the quarter-wavelength position. The simulated outlet acoustic pressure changed periodically with increasing standoff distance because of the standing wave characteristics in the acoustic transmission path. The peak outlet acoustic pressures appeared at standoff distances of approximately 6 mm and 22 mm, with corresponding simulated values of 5.65 × 108 Pa and 2.96 × 108 Pa, respectively.

**Figure 7:**
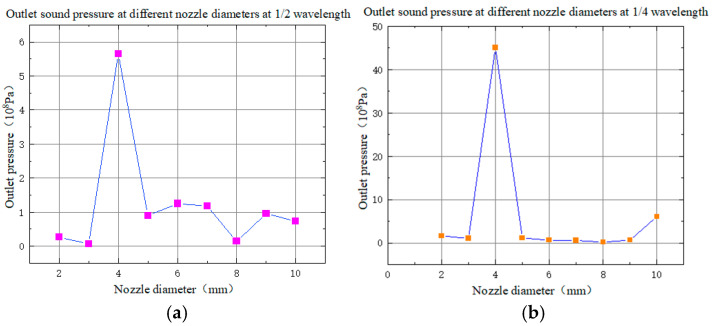
Pressure at different nozzle diameters: (**a**) λ_1/2_, (**b**) λ_1/4_.

The nozzle diameter also affected the outlet acoustic pressure, as illustrated in Figure 7. An excessively large nozzle diameter reduced acoustic energy concentration, whereas an excessively small nozzle diameter hindered acoustic transmission and abrasive-slurry discharge. When the standoff distance was fixed at 6 mm, the simulated outlet acoustic pressure reached a maximum at a nozzle diameter of 4 mm. Therefore, the acoustic simulation indicates that a nozzle diameter of 4 mm and a standoff distance close to the focused-pressure region are beneficial for acoustic energy concentration. It should be noted that the present COMSOL simulation only evaluates the acoustic pressure distribution in the FUAP nozzle assembly. It does not directly resolve cavitation-bubble collapse, abrasive particle collision, turbulent slurry flow, or brittle–plastic material removal.

## 3. Materials and Experimental Setup

### 3.1. Workpiece Material

**Figure 8:**
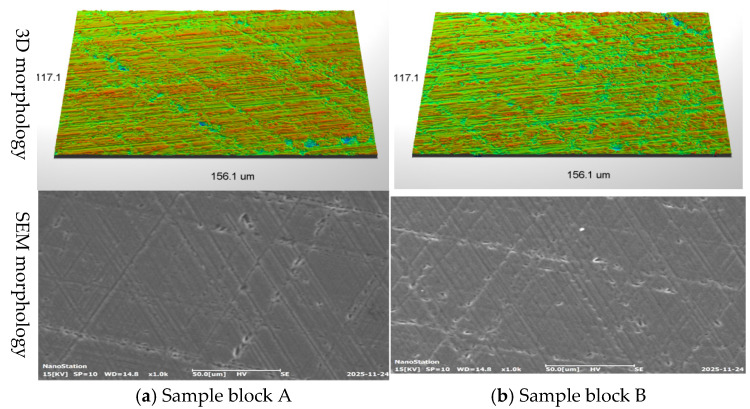
The initial surface state of the monocrystalline silicon experimental block.

Due to the anisotropic properties of monocrystalline silicon, the (111) crystal orientation possesses relatively strong mechanical properties compared with other orientations. Therefore, (111) oriented monocrystalline silicon was selected for polishing. The main physical and mechanical properties of the (111) orientation are shown in Table 2 [35,36]. The monocrystalline silicon samples were cylindrical blocks with dimensions of diameter × height = *φ*20 × 5 mm^3^. Before the polishing experiments, two sample blocks, A and B, were randomly selected for surface morphology inspection, as shown in Figure 8. The initial surface exhibited neat cutting textures, and the measured surface roughness *Ra* was approximately in the range of 0.16–0.22 μm.

### 3.2. Experimental Setup

**Figure 9:**
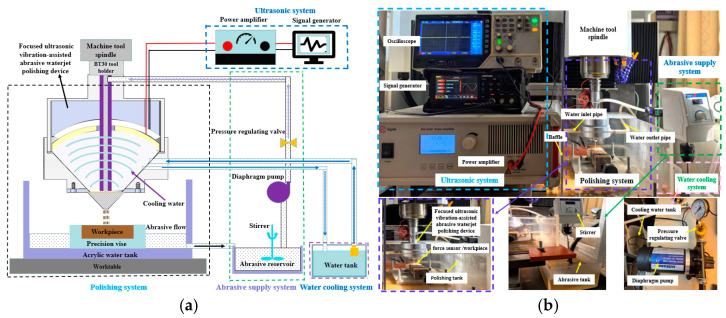
Focused ultrasound vibration-assisted abrasive waterjet polishing system: (**a**) schematic diagram (**b**) photograph.

A focused ultrasonic-assisted abrasive waterjet polishing system was constructed (Figure 9). It mainly consists of four subsystems: the ultrasonic system, the abrasive supply system, the polishing system, and the circulating water cooling system. These modules work together to control the entire polishing process. The ultrasonic system, serving as the vibration excitation core, comprises an ATA-6160(CN) power amplifier, a DG2052(CN) signal generator, a custom-designed focused ultrasonic vibration transducer, and an oscilloscope. The signal generator outputs a high-frequency excitation signal, which is amplified and power-compensated by the power amplifier, providing a total ultrasonic power supply of 425 W to drive the custom transducer to generate directional, focused ultrasonic vibration. The oscilloscope monitors the ultrasonic vibration signal in real time, enhancing the erosion and micro-cutting effectiveness of the abrasive waterjet.

**Figure 10:**
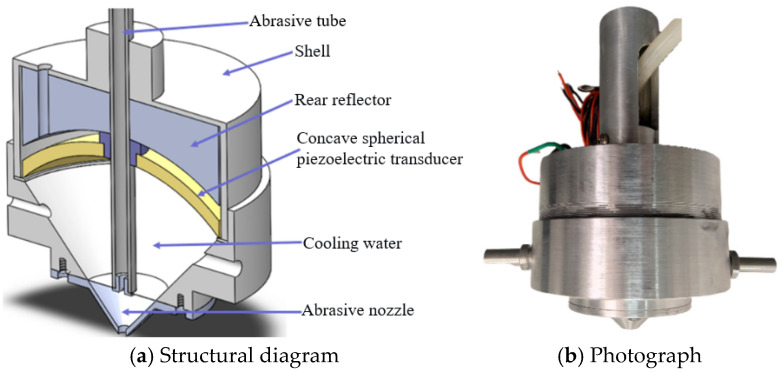
Structure of the focused ultrasonic polishing device.

To clarify the coupling mechanism between the focused ultrasound and the abrasive waterjet, the internal structure of the FUAP nozzle assembly is illustrated in Figure 10. The FUAP device consists of a concave spherical piezoelectric ultrasonic transducer, a rear reflector, a front conical chamber, a metallic diaphragm, and a front conical nozzle. A concave spherical reflector is installed in the rear holder. The spherical piezoelectric transducer is mounted in the holder by an elastic edge-supported structure. The gap between the convex surface of the transducer and the concave surface of the rear reflector acts as a rear matching layer, which improves the reflection of the vibration radiated from the back surface of the transducer. An acoustic impedance-matching material is arranged on the concave radiation surface of the piezoelectric transducer to improve the acoustic transmission from the transducer to the liquid medium. The concave radiation surface of the transducer, the front conical chamber, and the conical nozzle form a tapered internal space. This space is divided into upper and lower chambers by a metallic diaphragm located near the nozzle region. During operation, the upper chamber is filled with clean circulating water. The abrasive slurry is directly supplied into the lower conical chamber through a central feeding tube. Therefore, a continuous acoustic transmission path composed of clean water, the metallic diaphragm, and the abrasive slurry is formed inside the nozzle assembly. In this configuration, the focused ultrasonic wave is not coupled from air into a discontinuous free waterjet. Instead, ultrasonic vibration generated by the concave spherical piezoelectric transducer is transmitted through the clean water, metallic diaphragm, and abrasive slurry, and is focused near the nozzle region. The acoustic radiation force and cavitation effects are therefore introduced into the abrasive slurry before it is discharged from the nozzle. Under the combined action of focused ultrasonic energy and jet momentum, the abrasive slurry is ejected from the nozzle and impacts the monocrystalline silicon surface. This internal liquid–solid–liquid acoustic coupling path avoids the unrealistic assumption that ultrasonic waves must propagate through air and then enter the high-speed waterjet.

The polishing system integrates components such as the FUAP head, a high-precision force sensor, and a polishing water tank. Utilizing the positioning accuracy of the CNC machine tool’s worktable and the spindle feed precision, the distance between the abrasive jet nozzle and the workpiece surface, as well as the planar positioning and orientation of the workpiece, can be precisely controlled. This ensures accurate control of the polishing process and polishing pressure, guaranteeing the effectiveness of the focused ultrasonic-assisted abrasive waterjet polishing removal. To investigate the influence of the processing parameters of focused ultrasonic-assisted abrasive waterjet polishing on the machined surface quality and efficiency of monocrystalline silicon, a single-factor experimental method was employed. The initial jet velocity was calculated from the measured flow rate using *v*_0_ = *Q*/*A_n_*, where *Q* is the volumetric flow rate, and *A_n_* is the nozzle outlet area. The specific parameters are listed in Table 3. The abrasive concentration was defined as the mass fraction of abrasive particles in the polishing slurry. It was adjusted by changing the mass of abrasive particles added to the aqueous slurry while keeping the slurry preparation procedure consistent.

### 3.3. Characterization Methods

**Figure 11:**
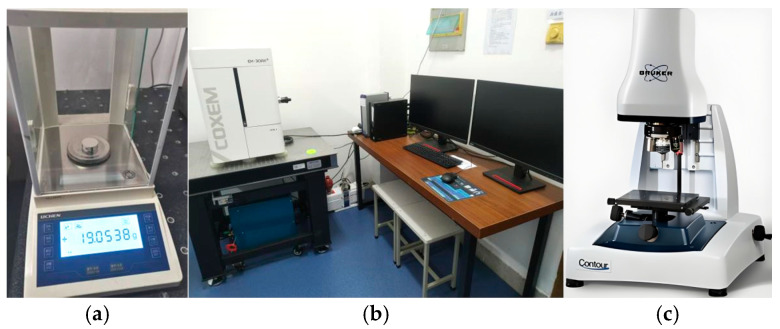
The polishing test section’s detection instruments: (**a**) precision balance; (**b**) SEM; (**c**) white light interferometer.

As shown in Figure 11, First, quantitative measurement of the material removal rate was conducted. The dried samples were placed on a precision electronic balance (FA2204N, Slade Limited, Shanghai, China) to accurately capture microscale material removal. Subsequently, the ultra-depth-of-field microscope (VHX-7000, Keyence, Osaka, Japan) and a Bruker ContourGT-K0 (Billerica, Massachusetts, US) white light interferometer were used in conjunction to perform three-dimensional surface morphology characterization and surface roughness parameter measurement of the polished samples. Finally, a scanning electron microscope (EM30plus, COXEM, Korea) was employed to observe the microstructure, defect distribution, and structural features of the polished surface.

## 4. Results and Discussion

### 4.1. Analysis of Material Removal Mechanism During Polishing of Monocrystalline Silicon

**Figure 12:**
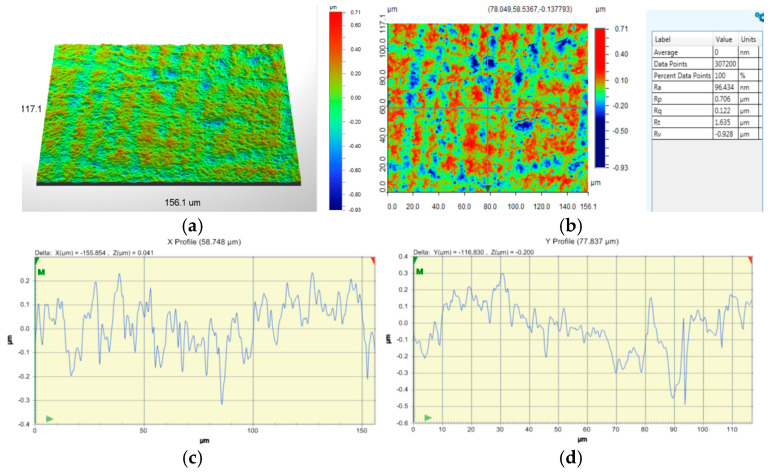
Surface condition of monocrystalline silicon after polishing: (**a**) three-dimensional morphology; (**b**) two-dimensional morphology; (**c**) X-axis contour; (**d**) Y-axis contour.

**Figure 13:**
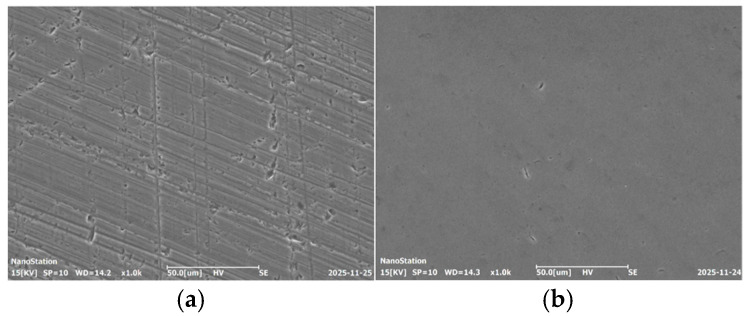
Surface morphology of polished monocrystalline silicon observed by SEM: (**a**) initial surface m (**b**) polished surface.

When the workpiece is monocrystalline silicon, a typical hard and brittle material, the initial surface morphology exhibits clear wire-saw cutting textures with a surface roughness Ra of 0.235 μm. After FUAP, due to the impact of abrasive particles and the erosion of ultrasonic cavitation micro-jets, the wire-saw cutting marks are essentially eliminated. The surface exhibits predominantly plastic removal traces (Figure 12), although some localized pits from brittle fracture are also present. The surface roughness Ra decreased to 0.096 μm, a reduction of approximately 59.1%. SEM inspection of the processed samples before and after polishing also reveals that the polished surface of the monocrystalline silicon shows improved cleanliness (Figure 13).

**Figure 14:**
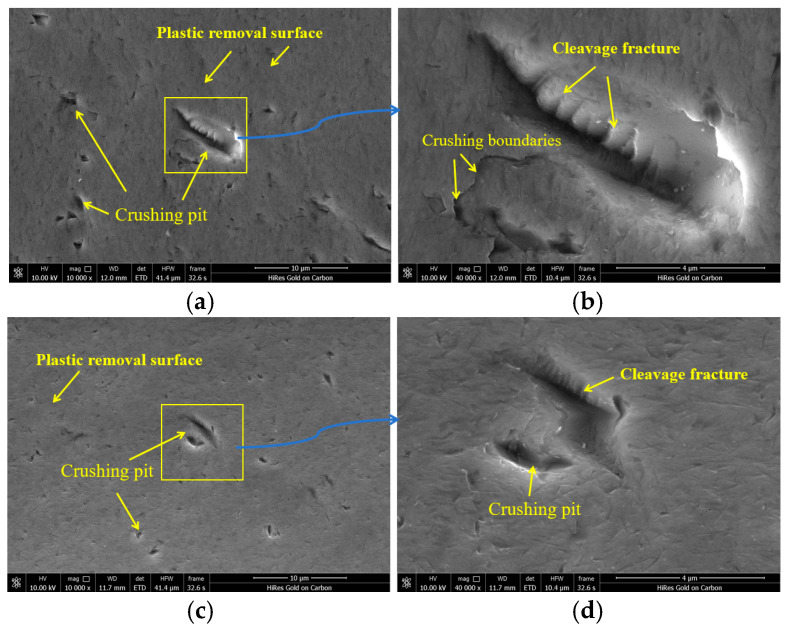
Typical surface morphology of the monocrystal silicon after polishing: (**a**) overall morphology without ultrasonic assistance; (**b**) local morphology without ultrasonic assistance; (**c**) overall morphology with ultrasonic assistance; (**d**) local morphology with ultrasonic assistance.

A precision balance was used to measure the material removal amount of the workpiece before and after polishing to evaluate removal effectiveness and compare it with the theoretical model established above. The results are shown in Figure 14. It was found that after FUAP, the material removal amount of monocrystalline silicon was approximately 2.91 mg/h, confirming the effectiveness of the polishing removal process. The focused ultrasonic field was assumed to be ideal, the sound intensity was considered uniform, and energy loss during ultrasonic transmission in water was neglected. In the actual polishing process, abrasive size dispersion, particle–particle collisions, jet turbulence, stochastic cavitation collapse, nonuniform abrasive concentration, and the coexistence of plastic chip formation and brittle fracture can all affect the real material removal amount. Therefore, the model should be regarded as a mechanism-oriented and semi-quantitative prediction model rather than a final precision prediction model for industrial ultra-precision polishing.

**Figure 15:**
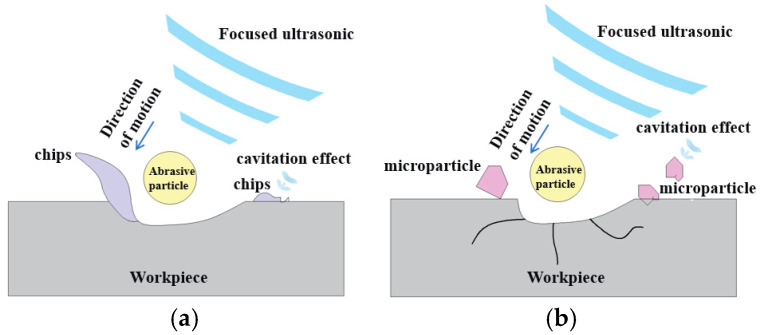
Schematic diagram of the material removal mechanism of focused ultrasound vibration AFP: (**a**) plastic removal; (**b**) brittle removal.

For the low-toughness, high-hardness monocrystalline silicon workpiece, the machined surface is mostly composed of smooth, flat areas accompanied by crushing pit defects (sized 3–7 μm). Further magnified SEM observation of local structures reveals stepped cleavage planes inside the crushing pits, which is a typical crushing morphology of brittle materials; local crushing boundaries are also clearly visible. Focused ultrasonic vibration provides not only acoustic field radiation force but also micro-jets and high-speed shock waves from cavitation. These factors act together on the monocrystalline silicon surface, increasing the material removal energy and making it more microscopic. Consequently, the removal effect on the hard and brittle silicon material at the microscale is improved, and the fracture removal phenomenon is suppressed. Based on the above analysis, the material removal mechanism during polishing under focused ultrasonic conditions is illustrated in Figure 15. The material removal behavior during FUAP should be interpreted as a mixed removal mode rather than a purely ductile-regime process. The smooth and flattened regions observed in the 3D morphology indicate that plastic shearing and chip formation occurred during polishing. However, the SEM images also show localized crushing pits with sizes of approximately 3–7 μm, together with stepped cleavage planes inside the pits. These features indicate that brittle fracture remained active in local regions. The calculated critical penetration depth of monocrystalline silicon was approximately 9.2 nm. Therefore, when the instantaneous penetration depth of abrasive particles exceeds this critical value, brittle cracks can be generated. Focused ultrasonic assistance can enhance abrasive particle motion and promote micro-cutting and plastic shearing, but it cannot completely eliminate brittle fracture. Therefore, FUAP of monocrystalline silicon involves the coexistence of plastic shearing/chip formation and localized brittle fracture. The role of focused ultrasound is to increase the contribution of plastic removal and reduce the severity of brittle-fracture defects, rather than to transform the process into fully ductile-regime removal.

### 4.2. Effect of Ultrasonic Power on Polishing Quality and Material Removal Rate of Monocrystalline Silicon

**Figure 16:**
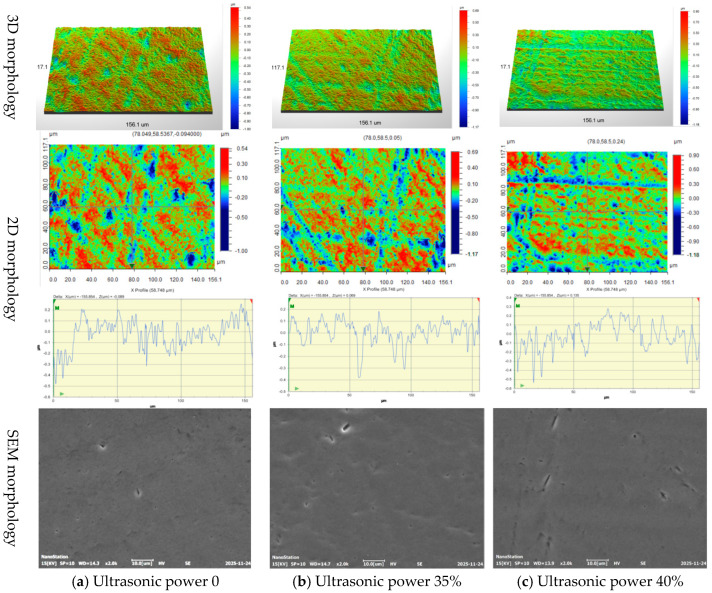
Surface morphology of monocrystalline silicon polished surface varies with the change in ultrasonic power. (**a**) Ultrasonic power 0 (**b**) Ultrasonic power 35% (**c**) Ultrasonic power 40%.

The ultrasonic vibration power directly affects the kinetic energy of abrasive particles impacting the workpiece surface. Figure 16 shows the influence of ultrasonic power on the surface morphology of polished monocrystalline silicon, with other parameters kept constant: abrasive size 1.5 μm, nozzle height 4 mm, processing time 16 min, abrasive concentration 10%, and jet angle 90°.

It can be observed that when the ultrasonic power is 0 (i.e., without ultrasonic-assisted polishing), the three-dimensional morphology (3D) shows clear initial abrasive scratches on the sample surface, with many wide and deep scratches (Figure 16a), consistent with the two-dimensional (2D) optical morphology and profile characteristics. Additionally, SEM images reveal microfracture pits caused by abrasive impact. When the ultrasonic power increases to 35% (Figure 16b), the kinetic energy of the abrasive particles increases, enhancing the material removal effect on the silicon surface. The 3D image shows weakened abrasive scratches and smaller grooves; the 2D image indicates reduced surface non-uniformity; however, the profile trace shows an increase in the maximum groove depth caused by abrasive impact, from 0.54 μm (without ultrasound) to 0.69 μm. The number of these extreme grooves is small, consistent with the SEM results. When the ultrasonic power is further increased to 40% (Figure 16c), the 3D image shows that the initial abrasive scratches on the silicon surface are almost completely eliminated.

**Figure 17:**
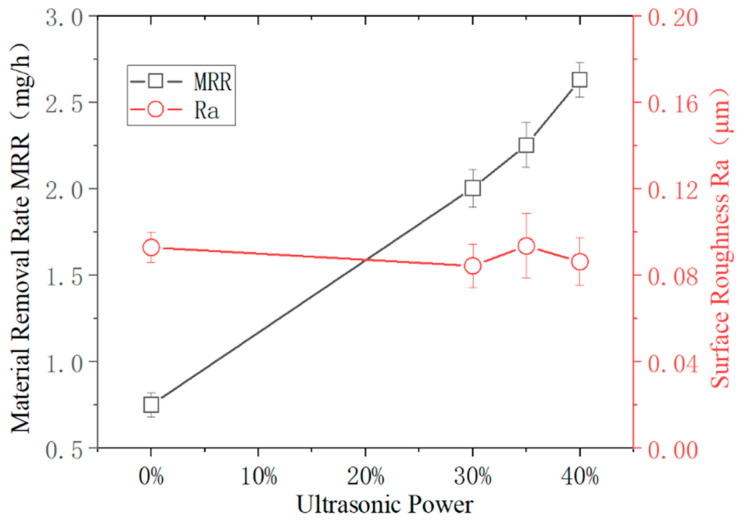
Effect of ultrasonic power on MRR and Ra.

Figure 17 illustrates the influence of ultrasonic power on the surface roughness and material removal rate of polished monocrystalline silicon. It can be seen that as the ultrasonic power increases from 0 to 40%, the surface roughness Ra varies between 0.0845 μm and 0.0937 μm, with a relatively small range of variation. Overall, Ra shows a relatively stable trend, indicating that under the current experimental conditions, the effect of ultrasonic power on surface roughness Ra is not significant. However, from the perspective of the material removal rate, increasing the ultrasonic power from 0 to 40% increases the MRR from 0.752 mg/h to 2.632 mg/h—approximately 3.5 times higher—showing a nearly linear growth trend. This suggests that as abrasive particles acquire more ultrasonic vibration energy, their impact kinetic energy is more substantial, leading to more efficient material removal from the silicon surface.

### 4.3. Effect of Abrasive Parameters on Polishing Quality and Material Removal Rate of Monocrystalline Silicon

#### 4.3.1. Abrasive Size

**Figure 18:**
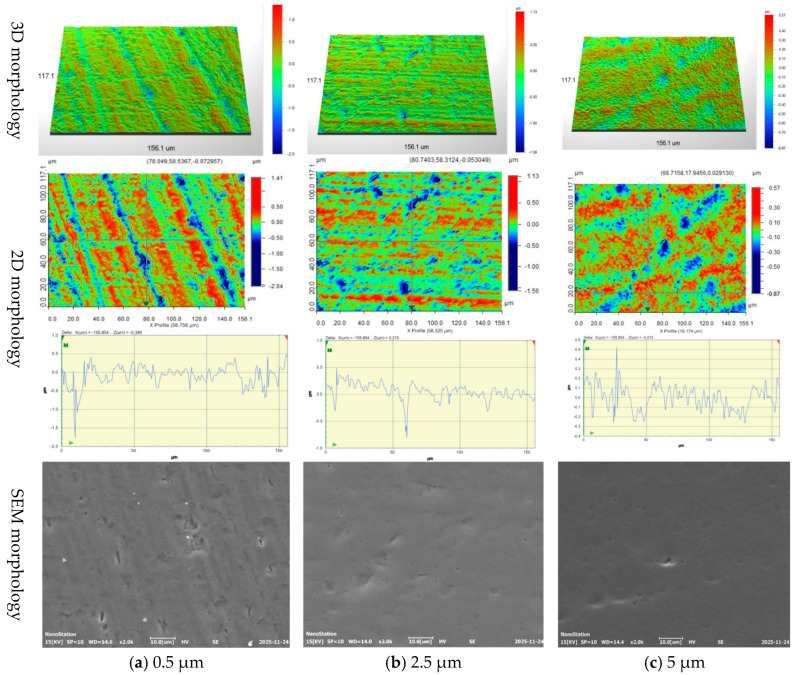
Surface morphology of monocrystalline silicon polished surface varies with the change in abrasive size. (**a**) 0.5 μm (**b**) 2.5 μm (**c**) 5 μm.

Figure 18 shows the influence of abrasive size on the surface morphology of polished monocrystalline silicon, with other parameters kept constant: ultrasonic power 30%, nozzle height 4 mm, processing time 16 min, abrasive concentration 10%, and jet angle 90°.

It was found that when the abrasive size is 0.5 μm (Figure 18a), the 3D morphology of the polished surface shows relatively clear initial abrasive scratches. Even after polishing, the maximum surface profile height reaches 1.41 μm, and SEM images reveal numerous surface defects, including ineffective removal of original abrasive scratches and pits formed by silicon fracture. These defects are somewhat improved when the abrasive size is 2.5 μm. However, the maximum profile height is still 1.13 μm (Figure 18b), only a 19.8% reduction compared with the 0.5 μm case. When the abrasive size increases to 5 μm (Figure 18c), the initial scratches from abrasive scratching are no longer visible in the 3D image, which shows micro-pits caused by abrasive impact; the texture after material removal is relatively uniform.

**Figure 19:**
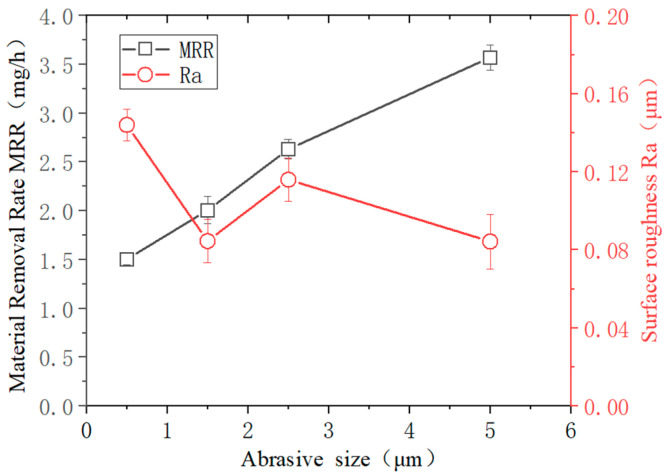
Effect of abrasive size on MRR and Ra.

Figure 19 shows the influence of abrasive size on the surface roughness and material removal rate of polished monocrystalline silicon. For surface roughness, when the abrasive size increases from 0.5 μm to 1.5 μm, Ra decreases from 0.1440 μm to 0.0845 μm, a reduction of 41.3%. As the size further increases to 2.5 μm, Ra increases to 0.1160 μm. When the size is 5 μm, the corresponding Ra is 0.0843 μm. Therefore, as the abrasive size varies from 0.5 μm to 5 μm, Ra decreases by approximately 41.4%, showing an overall fluctuating downward trend. Combined with the surface morphology analysis, it is evident that an appropriate abrasive size can increase the impact kinetic energy on the silicon surface, leading to better material removal and fewer surface defects.

#### 4.3.2. Abrasive Concentration

Abrasive concentration refers to the mass fraction of abrasive particles in the polishing slurry. In this study, the abrasive percentage was increased by adding more abrasive particles to the slurry while keeping the slurry preparation procedure consistent. Increasing abrasive concentration increases the number of abrasive particles transported to the focused ultrasonic energy region per unit volume of slurry. Therefore, the probability of particle impact, micro-cutting, and cavitation-assisted particle acceleration near the silicon surface is increased. As a result, material removal can be enhanced when the abrasive concentration is increased within an appropriate range. However, when the concentration is too high, particle agglomeration, mutual collision, shielding effects, and reduced slurry dispersion may occur. These effects decrease the proportion of abrasives effectively involved in polishing and may deteriorate surface quality. Therefore, investigating the influence of abrasive concentration on polishing quality and optimizing it is crucial.

**Figure 20:**
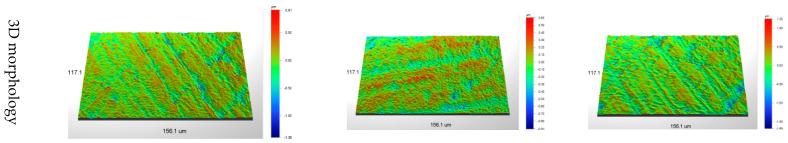

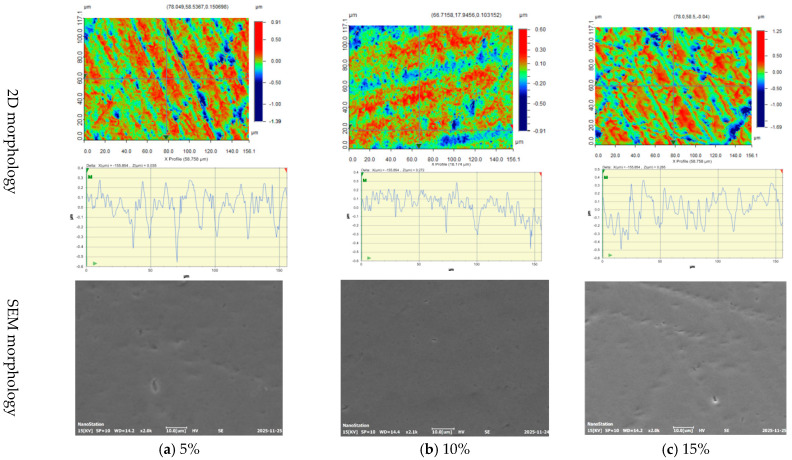
Surface morphology of monocrystalline silicon polished surface varies with the change in abrasive concentration.

The influence of abrasive concentration on the surface morphology of polished monocrystalline silicon is shown in Figure 20, with other parameters kept constant: ultrasonic power 30%, abrasive size 5 μm, nozzle height 4 mm, processing time 16 min, and jet angle 90°. The results show that at a concentration of 5%, the initial scratches on the silicon surface are somewhat eliminated, but some deep profiles remain (Figure 20a). The 2D image shows a maximum profile height of 0.91 μm, and the SEM image shows relatively few overall defects. When the concentration increases to 10%, the initial abrasive scratches are almost completely eliminated (Figure 20b); the material removal texture on the surface is relatively uniform and neat; the 2D profile shows a maximum height of only 0.60 μm, and the SEM image indicates good overall surface cleanliness. When the concentration reaches 15% (Figure 20c), the 3D image shows regions with apparent insufficient polishing, and the surface defects (SEM image) are residual traces of initial scratches; hence, the maximum profile height is larger (1.25 μm). Thus, under these composite polishing parameters, an abrasive concentration between 5% and 10% is beneficial for achieving good polishing quality on monocrystalline silicon.

**Figure 21:**
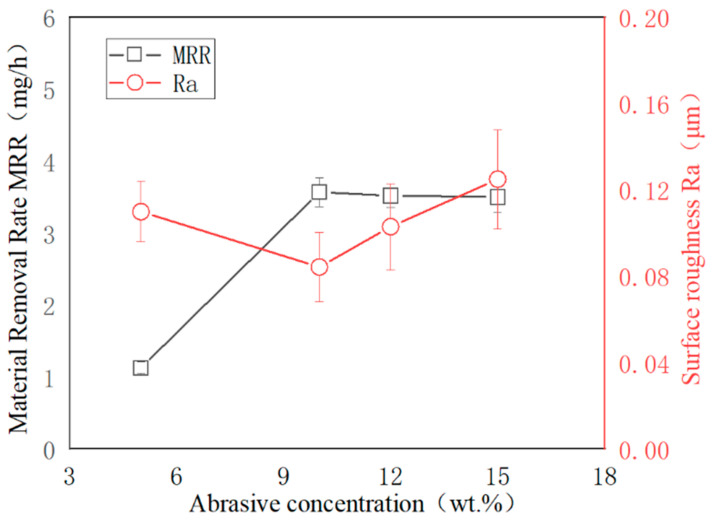
Effect of abrasive concentration on MRR and Ra.

Figure 21 shows the influence of abrasive concentration on surface roughness and material removal rate. At a concentration of 5%, the MRR is 1.1278 mg/h, and Ra is 0.1100 μm. When the concentration increases to 10%, the MRR increases to 3.5710 mg/h, corresponding to Ra 0.0843 μm—nearly three times the MRR at 5%, while Ra decreased by 23.3%. At a 12% concentration, the MRR and Ra are 3.5212 mg/h and 0.1030 μm, respectively. Compared with 10%, the MRR remains almost unchanged, but Ra increases by 22.1%. At 15%, the MRR is 3.5038 mg/h, and Ra is 0.125 μm. Compared with 12%, MRR decreases slightly, while Ra increases significantly by 11.3%. Overall, as the abrasive concentration increases from 5% to 15%, MRR first increases significantly and then stabilizes, while Ra first decreases and then shows an increasing trend. The increase in MRR from 1.1278 mg/h at 5 wt.% to 3.5710 mg/h at 10 wt.% can be attributed to the increased number of effective abrasive particles participating in impact and micro-cutting. At low concentration, the number of abrasives reaching the workpiece surface is insufficient, resulting in weak material removal and residual initial scratches. When the concentration increases to 10 wt.%, more particles are accelerated by the focused ultrasonic field and cavitation effect, leading to more frequent abrasive–surface interactions and a higher removal rate. However, when the concentration further increases to 12 wt.% and 15 wt.%, the MRR remains almost unchanged, while Ra increases. This indicates that an excessive abrasive concentration does not continuously increase the effective polishing action. Instead, particle agglomeration, particle–particle interference, and reduced dispersion stability may reduce the effective utilization ratio of abrasives and increase surface defects.

### 4.4. Effect of Abrasive Jet Angle on Polishing Quality and Material Removal Rate of Monocrystalline Silicon

**Figure 22:**
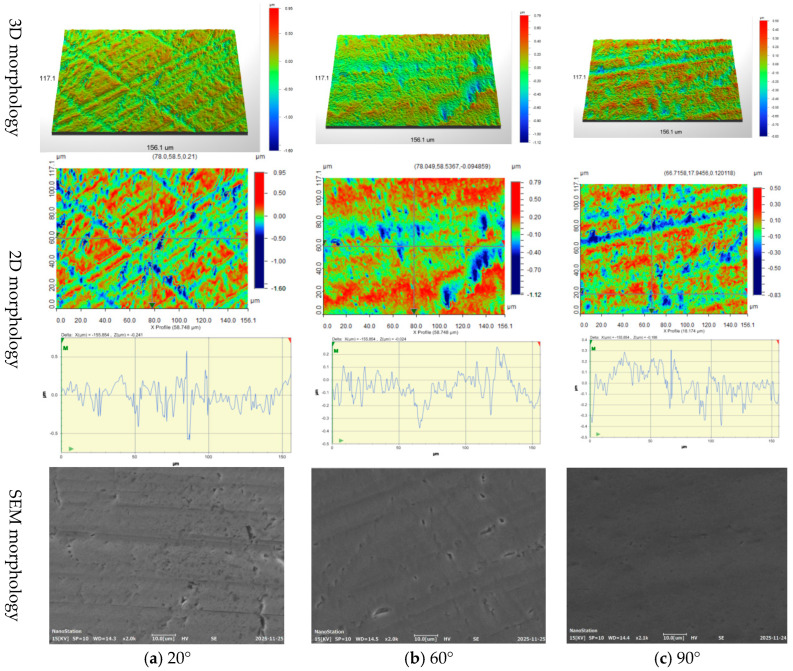
Surface morphology of monocrystalline silicon polished surface varies with the change in jet angle.

The influence of jet angle on the surface morphology of polished monocrystalline silicon is shown in Figure 22, with other parameters kept constant: ultrasonic power 30%, abrasive size 5 μm, nozzle height 4 mm, processing time 16 min, and abrasive concentration 10%. At a jet angle of 20°, many abrasive scratches and significant micro-pits from impact are observed (Figure 22a). When the angle increases to 60°, the initial scratches are basically eliminated, but some fracture pits caused by abrasive impact remain (Figure 22b), with a maximum profile height of 0.79 μm. At 90° (Figure 22c), the maximum profile height decreases to 0.50 μm.

**Figure 23:**
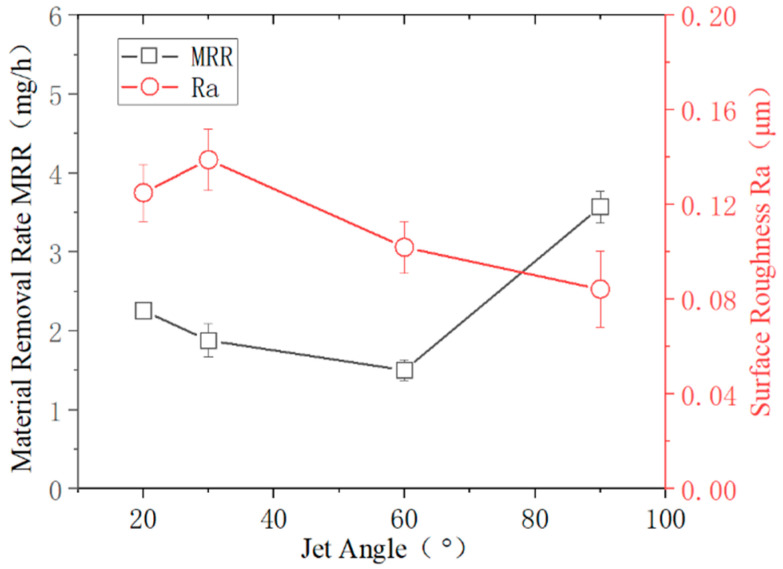
Effect of jet angle on MRR and Ra.

Figure 23 shows the effect of jet angle on surface roughness and material removal rate. When the angle increases from 20° to 30°, the MRR decreases from 2.2556 mg/h to 1.8797 mg/h, while Ra increases from 0.1250 μm to 0.1390 μm. A smaller jet angle results in lower impact kinetic energy for the particles, leading to greater scratching action on the silicon surface, more defects (as seen in Figure 23), and weaker material removal. As the angle increases from 30° to 60°, the MRR further decreases to 1.5038 mg/h, while Ra decreases to 0.102 μm. This is mainly because the increased jet angle enhances the cutting action of the particles on the silicon surface. Under the combined effect of plastic shearing and impact-induced microfracture removal, the initial scratches are mostly removed, reducing surface roughness. However, the impact kinetic energy remains relatively weak, so the MRR does not increase but slightly decreases. This situation improves significantly at a 90° angle, where the MRR increases to 3.571 mg/h, and Ra decreases to 0.0842 μm.

### 4.5. Effect of Nozzle Height

**Figure 24:**
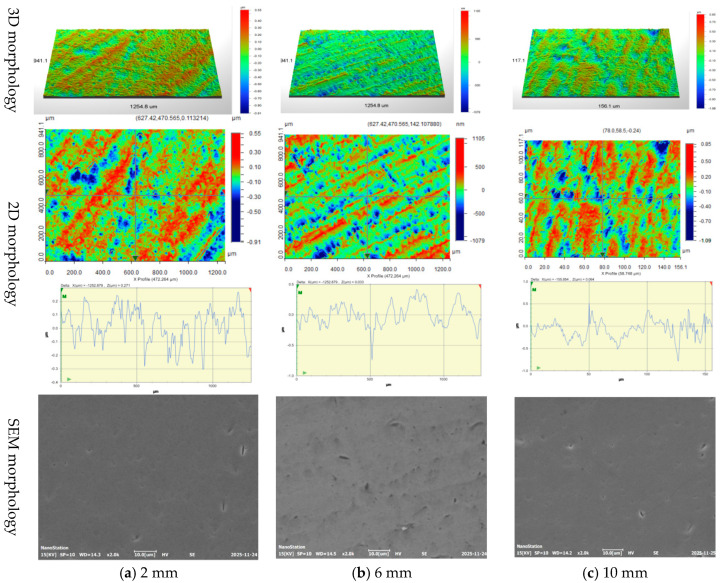
The surface morphology of the polished surface of single-crystal silicon varies with nozzle height.

The standoff distance determines the relative position between the workpiece surface and the focused ultrasonic energy region. Figure 24 shows the relationship between nozzle height and the surface topography of polished monocrystalline silicon, with all other parameters held constant. The results indicate that when the nozzle height is 2 mm (Figure 24a), the initial abrasive scratches on the monocrystalline silicon surface are effectively removed to a certain extent, and the minute, dense pits on the processed surface demonstrate the effect of abrasive impact on the monocrystalline silicon material under focused ultrasonic polishing conditions. The cross-sectional profile shows that the peak height of the polished surface at this time is approximately 0.55 μm, and the surface quality is good. Consequently, few defects are visible in the SEM morphology. This also indicates that at lower nozzle heights, the kinetic energy of the abrasive particles is sufficient to effectively remove material from the monocrystalline silicon surface without causing a large number of fracture defects. When the nozzle height was further increased to 6 mm (Figure 24b), the surface quality of the polished monocrystalline silicon deteriorated. The 3D topography revealed that numerous initial abrasive scratches remained on the workpiece surface after polishing, indicating that surface defects present in the initial state were not effectively removed. Furthermore, the maximum surface profile height under these conditions was 1.10 μm, and the polished surface exhibited numerous defects; consequently, the SEM images clearly reveal phenomena such as relatively large pits left behind after the removal of fractured monocrystalline material. When the nozzle height was 10 mm (Figure 24c), the nozzle was relatively far from the surface of the workpiece to be polished, and the abrasive particles failed to acquire sufficient kinetic energy to remove the monocrystalline silicon material. Consequently, the initial abrasive scratches on the monocrystalline silicon remained on the polished surface, resulting in reduced uniformity of the processed surface topography, with a maximum cross-sectional profile height of 0.85 μm. As mentioned earlier, the nozzle height primarily affects the focal point of the focused ultrasonic vibrational energy field. When the abrasive flow from the nozzle is precisely located within the focused ultrasonic action zone, the abrasive particles can gain greater kinetic energy. Consequently, when the nozzle height is between 2 and 6 mm, it is close to the focal region of the focused ultrasonic energy field, which promotes plastic chip formation during material removal. Conversely, when the nozzle height is between 6 and 10 mm, it is highly likely to be far from the focal region of the focused ultrasonic energy field.

**Figure 25:**
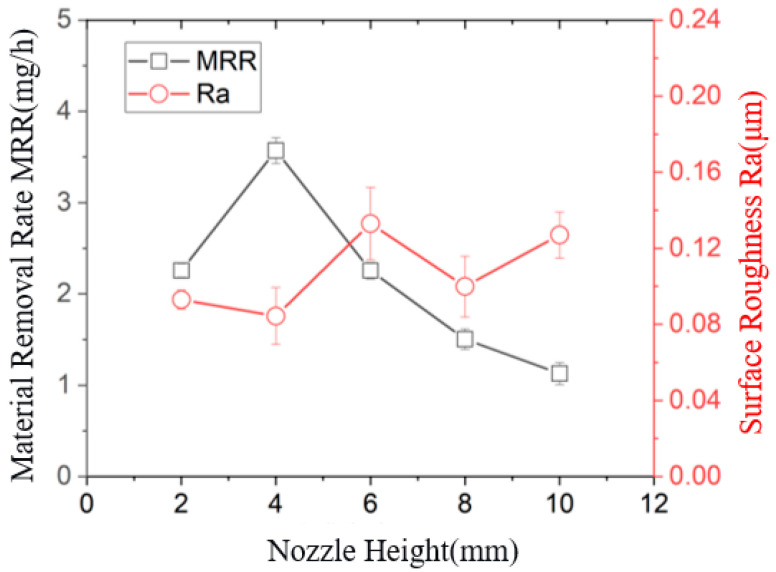
Effect of nozzle height on MRR and Ra.

Figure 25 further illustrates its effects on the surface roughness and material removal rate of polished monocrystalline silicon. As can be seen from the figure, surface roughness reaches its minimum value (Ra 0.0843 μm) when the nozzle height is 4 mm, and its maximum values (Ra 0.1330 μm and Ra 0.1270 μm, respectively) when the nozzle height is 6 mm and 10 mm. The effect of nozzle height on surface roughness exhibits a significant fluctuating trend; an appropriate nozzle height facilitates the combined effects of abrasive kinetic energy and ultrasonic cavitation within the focused ultrasonic vibration energy field. Regarding material removal rate (MRR), as the nozzle height increased from 2 mm to 10 mm, the MRR first rose from 2.256 mg/h to a maximum of 3.571 mg/h, then continued to decrease to 1.128 mg/h. It can thus be seen that this peak value represents the maximum material removal rate under the current experimental conditions (with a nozzle height of 4 mm). Therefore, it can be concluded that nozzle height not only influences the ratio of brittle-to-ductile removal when abrasive particles impact the material by controlling ultrasonic vibration energy but also plays a controlling role in the final machining quality and efficiency. When the distance is 4 mm, the workpiece surface is located within the effective focused ultrasonic energy region. Under these conditions, acoustic radiation and cavitation effects more effectively accelerate the abrasive particles, promoting the formation of plastic chips while suppressing excessive brittle fracture.

## 5. Conclusions

This paper analyzes the kinematics of abrasive particles under focused ultrasonic vibration conditions, establishes a material removal model for abrasive waterjet polishing under focused ultrasonic vibration, analyzes the material removal mechanism of brittle monocrystalline silicon during polishing, and verifies it experimentally. Furthermore, the effects of focused ultrasonic parameters and polishing process parameters on the material removal rate and workpiece surface quality were investigated, providing a fundamental theory and process technology for focused ultrasonic vibration abrasive jet polishing. The main conclusions are as follows:

(1) Kinematic analysis of abrasive particles under focused ultrasonic vibration conditions was conducted. The radiation force of the focused ultrasonic field and the focused ultrasonic cavitation forces (including high-speed micro-jets and intense shock waves) affecting particle velocity were studied, yielding an analytical mathematical expression. This expression comprehensively considers multiple influencing factors, including focused ultrasonic field radiation force, abrasive parameters, ultrasonic cavitation, and ultrasonic vibration parameters.
(2) A material removal model for focused ultrasonic vibration-assisted abrasive waterjet polishing of brittle monocrystalline silicon was established. This is a brittle material removal model based on crack evolution caused by high-speed particle impact, which considers factors such as abrasive parameters, particle motion parameters, and workpiece material properties. Verification experiments for the model were conducted.
(3) After FUAP, the material removal rate of monocrystalline silicon reached 2.91 mg/h under the representative condition. The surface roughness Ra was reduced by approximately 76.7%, confirming the feasibility of FUAP for improving material removal and surface quality. The COMSOL acoustic pressure simulation further confirmed that a localized focused ultrasonic pressure field was formed near the nozzle region, which provides a physical basis for the acoustic enhancement effect. However, the present simulation does not constitute a full quantitative prediction of material removal.
(4) The material removal process during FUAP of monocrystalline silicon involves the coexistence of plastic shearing/chip formation and localized brittle fracture. Comparison between the theoretical penetration depth of abrasive particles and this critical value indicates that purely ductile-regime removal cannot be assumed under all polishing conditions. The smooth regions on the polished surface suggest the occurrence of plastic shearing. Therefore, focused ultrasonic assistance promotes the contribution of plastic removal and mitigates brittle surface defects, but brittle fracture remains an important local removal mechanism. Due to the directional acoustic radiation force and ultrasonic cavitation effects of the focused ultrasonic energy field, compared with the single impact action of traditional waterjets, focused ultrasound superimposes alternating stress onto the impact. This mitigates microscale crushing pit defects during the brittle removal process of monocrystalline silicon, contributing to improved polishing outcomes.
(5) Appropriately increasing ultrasonic power, enlarging abrasive size, and raising abrasive concentration all contribute to enhanced material removal from monocrystalline silicon. Adjusting the nozzle height to the effective region of the focused ultrasonic energy field promotes material removal via chip formation while avoiding pit defects caused by excessive crushing. Optimal values for processing time and jet angle exist, which can enhance material removal efficiency while reducing scratch defects caused by abrasive impact.

## Figures and Tables

**Table 1:** The main physical and mechanical properties of monocrystalline silicon.

Material	Density kg/m^3^	Elastic Modulus GPa	Poisson’s Ratio	Hardness GPa	Yield Strength GPa	Fracture Toughness MPa·m^1^/^2^
Monocrystalline silicon (111)	2330	190	0.278	11.5	7	0.7

**Table 2:** Simulation parameter.

Component	Material	Parameter
Concave spherical piezoelectric Ceramic shell	PZT-8	Aperture radius *a* = 45 mm, thickness = 5 mm, Spherical radius of curvature *R*_0_ = 65 mm
Rear reflector block Rear matching layer Front matching layer	45 steel Air Silicone	Spherical radius *R_b_* = 69 mm Thickness = 4 mm
Metal diaphragm Ultrasonic parameters	316L	*d*_1_ = 0.1 mm *A* = 0.5 μm, *ƒ* = 430 kHz

**Table 3:** The composite polishing process parameters of the single-factor test for s monocrystalline silicon.

No.	Ultrasonic Power	Abrasive Size (μm)	Abrasive Concentration (wt.%)	Jet Angle (°)
1	0	1.5	10	90
2	30% (127.5 W)			
3	35% (148.75 W)			
4	40% (170 W)			
5	30% (127.5 W)	0.5		
6		1.5		
7		2.5		
8		5		
9			5	
10			10	
11			12	
12			15	
13			10	20
14				30
15				60
16				90

## Data Availability

The original contributions presented in this study are included in the article. Further inquiries can be directed to the corresponding authors.
